# Using Tibia Proximal Cut Autograft in Advanced Varus Knee Deformity in Total Knee Arthroplasty; Outcomes Compared to the Control Group

**DOI:** 10.2174/1874325001812010405

**Published:** 2018-10-24

**Authors:** Aydin Arslan

**Affiliations:** Elit Istanbul Medical Center, Orthopaedics and Traumatology, Istanbul Gelisim university, Istanbul, Turkey

**Keywords:** Knee, Arthroplasty, Varus, Tibial proximal, Total Knee arthroplasty (TKA), WOMAC

## Abstract

**Background:**

The purpose of this study was to compare the outcomes of Total Knee Arthroplasty (TKA) performed for advanced varus knee deformity, which is performed by using tibia proximal cut bone for the reconstruction of the tibia proximal medial bone defects, with a control group consisting of TKAs which did not need reconstruction.

**Methods:**

Patients in the present study underwent total knee arthroplasty between 2009 and 2015. 12 patients with advanced varus deformity who had undergone reconstruction with tibia proximal cut autograft and 15 patients who were randomly selected from patients who did not need reconstruction, were compared clinically and radiographically.

**Results:**

The mean follow-up period of the patients was found to be 73.1 ± 19.7 (36-108) months in the reconstruction group and 73.2 ± 12.3 (39-107) months in the control group. (*p*> 0.05) In both groups, significant improvement was observed postoperatively. In both groups, there was no evidence of loosening the required revision. WOMAC score was 32.4 ± 13.3 (8-64) in the reconstruction group and 28.9 ± 17.2 (6-70) in the control group at the last control visit. There was no difference between the groups when comparing the WOMAC scores at the last control visit. In the reconstruction group, the Hip-Knee-Ankle (HKA) angle was 26.1 ± 4.9 ° varus preoperatively and 1.3 ± 2.3 ° valgus postoperatively; and in the control group 10.1 ± 2.1 ° varus preoperatively and 2.7 ± 3.4 ° valgus postoperatively. (*p*> 0.05)

**Conclusion:**

In the present study, clinical and radiographic results of total knee arthroplasty patients, who suffered from advanced knee varus deformity and whose proximal tibia medial defects were reconstructed by using tibial proximal cut autograft, have been found to be successful when compared to the control group.

## 1 INTRODUCTION

In advanced varus deformed knees, the defect in the proximal medial tibia usually requires additional surgical procedures. [1-3] Methods used by surgeons for the reconstruction of this defect consist of providing support through cement, metal wedges, the use of autograft, *etc*. [1, 4-8] Although TKA has been known to be a satisfying surgery with a success rate above %90, in the case of advanced varus knee deformity, the outcomes of TKA is not clear. [9-11] We reconstructed the proximal medial defect by using proximal tibial cut bone to provide a firm support for the tibial base-plate.

## 2 MATERIALS AND METHODS

Patients in the present study underwent total knee arthroplasty due to primary osteoarthritis between 2009 and 2015. Patients who had undergone total knee arthroplasty due to rheumatoid arthritis, osteoarthritis secondary to trauma and metabolic diseases were excluded. 12 patients with advanced varus deformity (Hip-Knee-Ankle (HKA) angle >20° in all of 12 patients, >20° flexion contracture in 4 of 12 patients) who were reconstructed with tibia proximal cut autograft and 15 patients who were selected randomly from patients who did not need reconstruction, were compared clinically and radiographically.

Orthoroentgenograms were made during both the preoperative examination and the postoperative last control visit. HKA angle and tibia posterior slope angle were calculated in the preoperative examination and the postoperative control visit. Functional evaluation was performed with Western Ontario and McMaster Universities Osteoarthritis Index (WOMAC) in the preoperative examination and the postoperative last control visit. After the operation, patients were called on 15^th^ day, 45^th^ day, 3^rd^ month, 6^th^ month and one-year annual control visits. Patients were followed for at least 3 years. Preoperative BMI values of patients were measured.

A medial parapatellar approach was used in both the groups. Tibial proximal cut height was estimated as 10 mm below the joint line. Then, tibial medial defect was restored with saw. Lateral part of tibia proximal cut bone was used for reconstruction of the medial tibial defect. Tibia proximal cut bone was fixed with 2 cancellous screws. Fig. (**1**) demonstrates a 75 years-old patient with high-grade varus deformity intraoperative, preoperative and postoperative images) Cemented Posterior stabilized systems were used for all patients. (Zimed (Turkey), Sanatmetal (Hungarian), Ortopro (Turkey)) Ligament balancing was carried out in all cases. In particular, medial collateral ligament release was applied to achieve ligament balancing and suitable tibial insert sizes were chosen in each case. The patellar surface was not replaced. For infection prophylaxis, cefazolin IV 1 g was administered one hour before the operation. After the operation, ice compress was applied. Ankle pumping exercise was immediately performed. Isotonic and isometric knee exercises were started on day 1 postoperatively. After drains were removed, patients were mobilized using a walker on day 1 with full weight bearing. The patients were discharged after the fourth postoperative day. Subcutaneous 40 mg / 0.4ml enoxaparin sodium was started 12 hours after surgery for thromboembolism prophylaxis and continued once per day for three weeks.

### 2.1 Statistical Analyses

Statistical analyses were performed using SPSS, windows, version 21.0 (IBM Corp., Armonk, NY). Whether the data were normally distributed or not, was examined using the Shapiro-Wilk test. Comparisons between the groups were made using the Mann-Whitney U test. It was calculated that at least 12 cases should be taken from each group when α = 0.05 and 1-β (power) = 0.80 in the power analysis. A value of *p* <0.05 was considered statistically significant.

## 3 RESULTS

The mean age was 62.6 ± 7.4 (54-76) in the reconstruction group and 64.9 ± 9.3 (56-78) in the control group. The demographic characteristics, patient list and clinical and radiographic outcomes in detail are shown in Table **1**. The mean follow-up duration of the patients was 73.2 ± 15.1 (39-107) months in the reconstruction group and 67.1 ± 17.5 (36-108) months in the control group. (*p*> 0.05) In both groups, significant improvement was observed postoperatively. In the reconstruction group, WOMAC score was 32.4 ± 14.7 (8-64) in the last control visit and 28.9 ± 16.4 (6-70) in the control group. There was no difference when comparing the WOMAC scores of the groups at the last control visit. In the reconstruction group, the Hip-Knee-Ankle (HKA) angle was 26.1 ± 4.3 ° varus in the preoperative examination and 1.3 ± 2.4 ° valgus at postoperative last control visit; and in the control group 10.1 ± 3.3 ° varus in the preoperative examination and 2.7 ± 1.2 ° valgus at postoperative control visit. (*p*> 0.05) In the reconstruction group, the tibia posterior slope angle was 7.8 ± 3.1° in the preoperative examination and 8.5 ± 4.9° at postoperative last control visit. In the control group, the same angle was 9.1 ± 3.2° in the preoperative examination and 7.2 ± 5.7° at postoperative last control visit. Tibia posterior slope angles displayed no difference between the groups.

In the reconstruction group, one superficial infection occurred and was treated with an antibiotic treatment and dressing. In the control group, wound revision was performed in one patient due to a closure defect. Symptomatic DVT was not observed in the patients. There was no evidence of loosening that required revision in both groups. There was no subsidence of autograft in the reconstruction group.

## 4 DISCUSSION

It is not preferable that the proximal tibia cut level is lower than accepted measures. [1, 6, 12] That is because the tibial base plate cannot be supported by softer cancellous bone and the tendons and ligaments adhering to the proximal tibia may be damaged [1, 6]. For this reason, most of the defects of tibial medial plateau have to be reconstructed. [6] Lee and Choi [8] used metal rectangular augments and reported successful outcomes at a minimum 5 year follow up. Reportedly, there is no difference between metal rectangular augments and metal wedges. [13] Besides, downsizing and lateral shift of tibial base plate were reported as successful in long-term follow up. [14, 15] However, it has been reported that the cheapest and most convenient way to reconstruct this defect is using autografts. [1, 5, 16] It has been shown that autograft is incorporated with creeping substitution [17].

It has been reported that iliac crest grafts, femoral and tibia cut autografts were used to biologically reconstruct the tibia medial defect. [1, 2, 5, 6, 18-21] We used autografts obtained from tibia cuts in the reconstruction of these defects. This was due to the fact that iliac crest graft harvesting causes morbidity in donor site and that proximal tibia cut autograft is more resistant than the femoral condyle cut autografts.

In previous studies, patients with varus deformities > 15 degrees were biologically reconstructed using tibia cut and femoral cut autografts. Besides that, cortical screws were used for fixation, similar to the method in the present study. [1, 5, 6] In this study, functional and radiological outcomes of the reconstruction group were not different from the control group and the outcomes of both groups were similar when compared with the previous studies. [1, 5, 6, 22] There was no evidence of component loosening requiring revision. In the present study, HKA angle was reconstructed successfully when compared with the control group and previous studies. [1, 5, 6, 23, 24] Different from the previous studies, no stem extensions were used in the reconstruction group in the present study. Complication rates were lower than the previous studies. [5, 6, 10, 17-20] In this study, there were two major limitations. These were the small number of patients in the group requiring reconstruction and the need for a longer follow-up for all patients.

## CONCLUSION

In the present study, clinical and radiographic results of total knee arthroplasty patients, who suffered from advanced knee varus deformity and whose proximal tibia medial defects were reconstructed by using tibial proximal cut autograft, have been found to be successful when compared to the control group.

## Figures and Tables

**Fig. (1):**
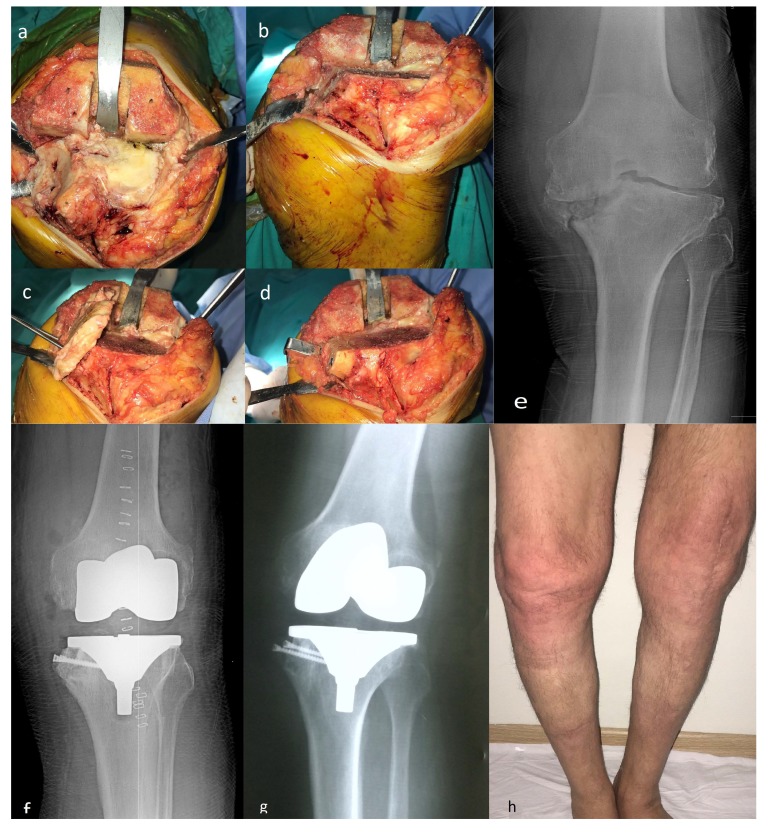
Intraoperative photographs of 75 years old patient’s left knee, **a**; tibia medial plato defect is seen, **b**; after tibia proximal cut medial side is prepared for fixation, **c**; tibial cut autograft is fixed, **d**; autograft is cut, **e**; preoperative radiographic image, **f**; postoperative immediate radiographic image, **g**; first year control visit radiographic image, **h**; first year photo of lower extremity.

**Table 1:** Demographic features, patient list and outcomes of the groups in detail.

**Reconstruction Group**							
Patient no	Gender	Age (year)	BMI (kg/m2)	Follow Up Time (Month)	Womac Scores	HKA Angle Preop (Varus)	HKA Angle Post Op (Valgus)
1	M	59	33,2	71	22	22	3
2	F	75	33,7	72	22	31	-3
3	M	56	28,6	39	8	33	-1
4	F	65	30,1	72	26	20	2
5	F	67	32,3	80	33	24	2
6	F	57	26,8	73	29	26	1
7	M	62	31,6	63	44	23	2
8	F	54	34,1	78	40	29	4
9	M	55	33	107	45	26	-2
10	M	65	34,7	73	64	21	5
11	F	59	31,9	77	36	27	3
12	F	76	34,8	73	20	31	0
**Control group**							
Patient no	Gender	Age (year)	BMI (kg/m2)	Follow Up Time (Month)	Womac Scores	HKA Angle Preop (Varus)	HKA Angle Post Op (Valgus)
1	F	77	31,2	66	18	12	4
2	F	78	28,6	78	17	13	1
3	F	56	31,8	65	19	8	3
4	F	67	27,2	65	16	8	4
5	M	77	26,2	108	35	9	4
6	F	62	26,3	66	36	11	1
7	F	57	31,6	78	43	5	3
8	M	76	32,7	78	70	8	4
9	M	57	31,8	47	37	8	2
10	M	58	34,7	73	50	9	4
11	F	57	31,3	77	6	9	2
12	F	77	32,1	73	26	19	3
13	F	59	24,5	36	18	13	1
14	F	56	32,3	43	22	10	2
15	F	59	26,3	54	21	9	3
